# Editorial: Environmental Monitoring and Remediation Using Microbiotechnology

**DOI:** 10.3389/fmicb.2022.927867

**Published:** 2022-05-13

**Authors:** Tian Li, Lean Zhou, Xiaojing Li, Li Yuan, Wei Zhi

**Affiliations:** ^1^Ministry of Education (MOE) Key Laboratory of Pollution Processes and Environmental Criteria/Tianjin Key Laboratory of Environmental Remediation and Pollution Control/College of Environmental Science and Engineering, Nankai University, Tianjin, China; ^2^School of Hydraulic and Environmental Engineering, Changsha University of Science and Technology, Changsha, China; ^3^Agro-Environmental Protection Institute, Ministry of Agriculture and Rural Affairs/Key Laboratory of Original Agro-Environmental Pollution Prevention and Control, Ministry of Agriculture and Rural Affairs (MARA)/Tianjin Key Laboratory of Agro-Environment and Agro-Product Safety, Tianjin, China; ^4^Chinese Academy of Sciences (CAS) Key Laboratory of Urban Pollutant Conversion, Department of Environmental Science and Engineering, University of Science and Technology of China, Hefei, China; ^5^Department of Civil and Environmental Engineering, Pennsylvania State University, University Park, PA, United States

**Keywords:** environmental monitoring, environmental remediation, microbiotechnology, wastewater treatment, modeling design

With the continuous improvement of the level of scientific and technological innovation, many emerging materials and chemicals have come into our lives, which has brought great pressure to the environment and caused an increasing number of contamination problems (Atashgahi et al., 2018; Escher Beate et al., 2020; Johnson Andrew et al., 2020). Microbiotechnology is considered to be environmentally friendly and sustainable when dealing with contaminants and has been attracting tremendous attention (Ahmed et al., 2021; Li et al., 2021, 2022b). Considering the degradation period of contaminants and the mechanism conducted by microbes, oxidation and reduction reactions occur all the time, providing the most basic support for early warning and remediation using microbiotechnology (Li et al., 2018, 2022a; Zhang et al., 2021). The appearance of contaminants is bound to break the balance of the original ecological environment, so how to monitor the existence of contaminants online, *in situ*, and quickly will become one of the key issues in the early warning field, and how to realize the efficient degradation of contaminants by microbes will become one of the key difficulties in remediation field.

In this special issue, we set up the Research Topic of *Environmental Monitoring and Remediation Using Microbiotechnology* in the journal of *Front. Microbiol*., which had attracted a lot of attention from researchers. This topic mainly focuses on the monitoring and remediation of contaminants in water, sediment, and soil using microbes and the mechanisms of the interaction between microbes and the surrounding environment. For early warning of contamination using microbes, various signals can be collected: bioelectrical signals through electron transfer, biochemical signals through material coupling, or biophysical signals through matrix changes. As for the remediation using microbes, pure bioremediation, phyto-bioremediation, electrically/chemically driven bioremediation and other improved bioremediations are well described. The continuous challenge is to design the processes to include nutrient recovery, which will be aided by further exploration through the interface between microbes and contaminants.

Specifically, 18 papers were accepted and published on this Research Topic, which was contributed by 118 authors from around the world. The papers that were viewed more than 2,000 times were from *Transcriptome-Guided Insights Into Plastic Degradation by the Marine Bacterium* contributed by Kumari et al., *Advances in Studies on Microbiota Involved in Nitrogen Removal Processes and Their Applications in Wastewater Treatment* contributed by Mai et al. and *An Ultrafast One-Step Quantitative Reverse Transcription–Polymerase Chain Reaction Assay for Detection of SARS-CoV-2* contributed by Milosevic et al. The research directions of these papers were the hot spot in the current environment. The first paper focused on microplastic degradation by microorganisms. Through the detection of PET hydrolysates, the genes of degraded PET were identified and cloned as an environmentally friendly solution to improve the utilization of PET by microbial systems. The second paper reviewed the microflora, pathways and related functional genes involved in nitrogen removal, and discussed the methods to improve nitrogen removal efficiency in the microbial treatment of industrial wastewater (Mai et al.). The third paper developed an ultrafast one-step RT-qPCR assay for SARS-CoV-2 detection, which significantly reduces the running time of conventional RT-qPCR (Milosevic et al.). Meanwhile, other papers also included the studies on modeling design, pollutant degradation and greenhouse gas emissions, which also covered water, soil and constructed wetlands involved with microorganisms. There was only one review paper among the remaining 15 papers, entitled *A Review on Microorganisms in Constructed Wetlands for Typical Pollutant Removal: Species, Function, and Diversity*. In this review, an in-depth bibliometric analysis of microbial studies in constructed wetlands (CWs) was performed to evaluate research trends and identify the most studied pollutants, which could provide new ideas and directions for the research of microorganisms in CWs (Wang et al.).

Four papers focus on bacterial isolates against different contaminants and harmful substances, including atrazine, Nitrogen, *Karenia mikimotoi*, and Roxarsone. In the study of Jia et al., *Paenarthrobacter* sp. AT-5, an atrazine-degrading strain, was inoculated into agricultural soils contaminated with atrazine to investigate the bioaugmentation process and the reassembly of the soil microbiome. They reported that the inoculation of strain AT-5 significantly affected the community structure of the soil microbiome, and the abundances of bacteria associated with atrazine degradation were improved (Jia et al.). Sun et al. prepared biochar/clay composite particle (BCCP) as the carrier to immobilize *Ochrobactrum* sp. to consume ammonium nitrogen (${\text{NH}}_{4}^{+}$-N), and the effects of the calcined program and immobilizing material were investigated. Ding et al. investigated the biochemical and physiological responses of *K. mikimotoi* to the algicidal bacterium *Paracoccus homiensis* O-4. The effects on the levels of reactive oxygen species (ROS), malondialdehyde content, multiple antioxidant systems and metabolites, photosynthetic pigments, and photosynthetic index were also examined. This research provides insights into the prophylaxis and control of harmful algal blooms *via* interactions between harmful algae and algicidal bacteria (Ding et al.). In the study of Li W. et al., wheat-straw-derived biochar was used to investigate how biochar amendment affected *Shewanella oneidensis* MR-1 growth and roxarsone transformation in water under anaerobic conditions. Their results suggested that wheat-straw-derived biochar may be an important agent for activating microbial growth and can be used to accelerate the transformation of roxarsone, which could be a novel strategy for roxarsone remediation (Li W. et al.). In addition to studies of pure bacteria, there are also studies of mixed bacteria. Mao et al. assessed the microbiological effects of black carbon (BC) by using a fluorescent fingerprinting assay based on flow cytometry (FCM) of bacterial communities with low (LNA) and high (HNA) nucleic acid-content bacteria. They also investigated a high-resolution temporal variation of bacterial abundance and LNA/HNA ratio in Tibetan ice cores and revealed that bacterial abundance was proportional to the atmospheric BC on the glaciers. In the study of Song et al., the biodiversity and functional characteristics of microplastic-attached biofilms originating from two freshwater bacterial communities were reported. The results from 16S rRNA amplicon sequencing showed that the dynamic biofilm successions on different microplastics were highly dissimilar. This study also speculated that more symbionts and parasites colonized microplastics in the tap water than in the lake water (Song et al.). Fungal research is also reflected in this special issue. Zhou et al. demonstrated the effect of Arbuscular mycorrhizal fungi (AMF) on the uptake and transport of Stibium (Sb) in the soil–rice system, facilitating future research on the related mechanism in the soil–rice system under Sb stress. Zhong et al. reported that fungi were dominant in N_2_O production processes followed by archaea in Northern Chinese grasslands and the key variables of N_2_O production and the nitrogen (N) cycle depended on the dominant microbial functional groups in the N-cycle in soils. Moreover, the study of Liu et al. explored the effects of environmental factors on the longitudinal plankton patterns, through a 5-year-long study on the environmental factors and communities of phytoplankton and zooplankton in an alpine cascade reservoir system located upstream of the Yellow River region.

Different pollutants are also discussed as research priorities, including chemical oxygen demand (COD), polycyclic aromatic hydrocarbons (PAHs), crystal violet, methylene blue, etc. In the research of Huang et al., a mathematical simulation model was established to investigate the performance of a full-scale anaerobic biochemical system for treating the COD in deinking pulp wastewater. Wang et al. investigated the adsorption mechanism of crystal violet and methylene blue and performed the extraction of activated carbon (AC) and AC-based ZVI by solid-phase and liquid-phase reduced approaches. Huang et al. explored the performance of denitrification deep-bed filter (DN-DBF) to treat municipal sewage and investigated the metabolic pathway for meeting a more stringent discharge standard of total nitrogen (TN). Li Y. et al. explained the influence mechanism of soil salinity on PAH biodegradation from the perspective of degradation genes and soil enzyme activities. In the study of Guo et al., a colorimetric enzyme biosensor was developed for one-step detection of hypoxanthine (Hx), which provided a robust advantage in the economic reaction system, ease of preparation, short time consumption, and moderate reaction temperature compared with other methods.

With the emergence of new pollutants and the continuous improvement of environmental standards, environmental pollution monitoring and remediation will be paid more and more attention. How to realize *in-situ* monitoring and efficient remediation will be a problem worthy of attention in the future.

## Author Contributions

TL wrote this editorial note. All authors edited the final text and contributed to the article and approved the submitted version.

## Conflict of Interest

The authors declare that the research was conducted in the absence of any commercial or financial relationships that could be construed as a potential conflict of interest.

## Publisher's Note

All claims expressed in this article are solely those of the authors and do not necessarily represent those of their affiliated organizations, or those of the publisher, the editors and the reviewers. Any product that may be evaluated in this article, or claim that may be made by its manufacturer, is not guaranteed or endorsed by the publisher.
